# 

**Published:** 1897-09-18

**Authors:** 


					THE HOSPITAL. ABSOLUTELY PURE, therefore BEST. Sept. 18th, 1897.
> *
S. X,
CADBURY'S -ajA, ? w- CADBURY^S 5 .
cocoa IT fHl[ mr* cocbftnaeor; ccneraeisoi
" The standard of highest purity at preient 1^, Jfg* ^ NO ALKALIES USED., r. ~
attainable. Lantel, S E P, ~27,' 1897
No. 573. Vol. XXII. Sattopay,
Sept. 18th, 1897.
[SuhscnptionTorms,] pRICE TWOPENCE.

				

